# Connectomics of bipolar disorder: a critical review, and evidence for dynamic instabilities within interoceptive networks

**DOI:** 10.1038/s41380-018-0267-2

**Published:** 2018-10-02

**Authors:** Alistair Perry, Gloria Roberts, Philip B. Mitchell, Michael Breakspear

**Affiliations:** 1 QIMR Berghofer Medical Research Institute, Brisbane, QLD Australia; 2 Max Planck UCL Centre for Computational Psychiatry and Ageing Research, Berlin/London, Germany; 3 Center for Lifespan Psychology, Max Planck Institute for Human Development, Lentzeallee 94, 14195 Berlin, Germany; 4 School of Psychiatry, University of New South Wales, Randwick, NSW Australia; 5 Black Dog Institute, Prince of Wales Hospital, Randwick, NSW Australia; 6 Metro North Mental Health Service, Brisbane, QLD Australia

**Keywords:** Neuroscience, Bipolar disorder

## Abstract

The notion that specific cognitive and emotional processes arise from functionally distinct brain regions has lately shifted toward a connectivity-based approach that emphasizes the role of network-mediated integration across regions. The clinical neurosciences have likewise shifted from a predominantly lesion-based approach to a connectomic paradigm—framing disorders as diverse as stroke, schizophrenia (SCZ), and dementia as “dysconnection syndromes”. Here we position bipolar disorder (BD) within this paradigm. We first summarise the disruptions in structural, functional and effective connectivity that have been documented in BD. Not surprisingly, these disturbances show a preferential impact on circuits that support emotional processes, cognitive control and executive functions. Those at high risk (HR) for BD also show patterns of connectivity that differ from both matched control populations and those with BD, and which may thus speak to neurobiological markers of both risk and resilience. We highlight research fields that aim to link brain network disturbances to the phenotype of BD, including the study of large-scale brain dynamics, the principles of network stability and control, and the study of interoception (the perception of physiological states). Together, these findings suggest that the affective dysregulation of BD arises from dynamic instabilities in interoceptive circuits which subsequently impact on fear circuitry and cognitive control systems. We describe the resulting disturbance as a “psychosis of interoception”.

## Introduction

Bipolar disorder (BD) is a relatively common disorder with a substantial illness burden and high risk of suicide [1, 2]. The classic picture of BD is of a relapsing/remitting condition with relatively brief elevations in mood followed by protracted episodes of depression [1, 3]. However, the natural history, response to treatment and clinical presentation of BD are quite heterogeneous. Brief instances of elevated mood early in the disorder may be overlooked, such that treatment focuses on the initial depressive episodes [4–6], despite the presence of an underlying disorder that may be better managed with mood stabilizers than antidepressants [6–9]. For these reasons, the clinical diagnosis of BD may be substantially delayed until the episodic and bivalent nature of the illness has clearly expressed itself [10]. Problematically, the efficacy of pharmacological treatments in BD is dependent on an accurate and early diagnosis [7]. These issues underline the need for a better understanding of the neurobiology of BD and, crucially, the development of biomarkers that are present early in the disorder.

Fluctuations in mood, affect, and motivation are a cornerstone of human experience, allowing us to anticipate and adjust our social interactions according to context [11]. In BD, such fluctuations become sufficiently pronounced, pervasive and persistent to cause distress and functional impairment [12]. Contemporary neurobiological theories of emotion posit a constellation of fronto-limbic regions (e.g. the hippocampus and insula), their connections to anxiety and fear circuitry (i.e. amygdala), and their interactions with regions traditionally implicated in cognitive control (such as the inferior frontal gyrus [IFG] and anterior cingulate cortex [ACC]) [13, 14]. Corresponding models of BD propose that dysfunction in these fronto-limbic neural circuits underlies the emotional and cognitive dysregulation that characterise the disorder [15, 16]. Traditionally, these models find support in structural magnetic resonance imaging (sMRI) findings of morphological abnormalities in fronto-limbic and subcortical structures [17–19]. Similarly, functional MRI (fMRI) studies of BD have consistently reported over-activation in the amygdala and other limbic structures during emotional processing and regulation [16, 20, 21]. Recent studies of high risk cohorts likewise report functional and morphological differences in functionally related regions [15, 22, 23].

Classic theories of brain function focus upon cognitive and emotional function in segregated, functionally specialized regions [24]. The notion that BD arises from dysfunction within regions supporting emotion regulation sits within this framework. To this body of knowledge, recent research has added the integrative role of large-scale circuits and networks in health [25–27] and disease [28, 29]. Patterns of anatomical wiring are organized into networks [30–32] that shape dynamic patterns of large-scale neural activity [33–35]. The integration of sensory, associative and motor areas into brain networks supports the complex features of human cognition and behavior, which cut across systems and modalities [36]. Accordingly, many psychiatric conditions have been positioned as reflecting dysfunction amongst these large-scale interactions, resonating with Geschwind’s earlier dysconnectionist school [28, 37]. Schizophrenia (SCZ) is the classic dysconnection syndrome—with mistimed network activity [29, 38, 39] resulting in the dysfunction of synaptic plasticity and learning [40, 41]. Likewise the symptoms of neurodegenerative disorders have been framed as reflecting loss of neural integration within large-scale cortical networks [42] and functionally integrated circuits [43].

In this paper, we view BD through the prism of connectomics. We first review the basic definitions of brain connectivity theory and their application to neuroimaging data. We then highlight the body of work on structural, functional and effective connectivity disturbances in BD and high risk (HR) cohorts. We finally discuss recent developments in connectomics and computational neuroscience more broadly: we use this body of work to propose that BD reflects a loss of stability in large-scale brain network dynamics, and more specifically, those which subserve physiological homeostasis and interoception (the perception of physiological states).

## Brain networks in health and illness

**Fig. 1:**
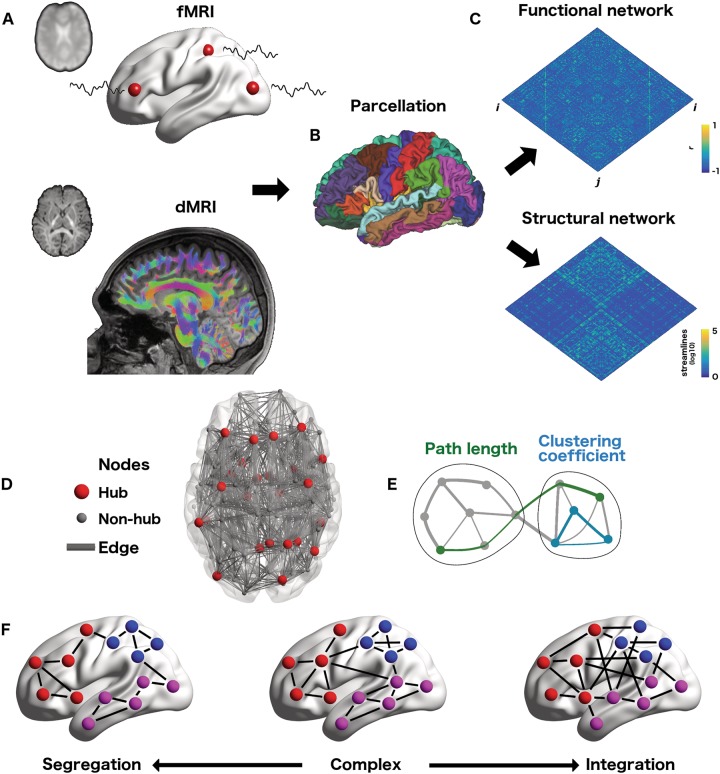
Fundamentals of connectomics: **a** Construction of functional and structural networks. *Top*: Correlated BOLD fluctuations (black curves) in grey matter regions (red spheres) derived from resting state fMRI acquisitions form the basis of functional connectivity. *Bottom*: Fibre tracts, reconstructed from the dMRI data, form the basis of whole brain structural connectivity. **b** To create a network representation of these data, grey matter is parcellated into a number (*N*) of discrete and bounded regions (nodes). These boundaries can derive from atlases, anatomical, histological or even connectivity criteria. Ideally all nodes should be of approximately equal size and surface area, and define functionally homogenous grey matter tissue. **c** Combining the functional and/or structural connectivity data with the parcellation yields functional and structural connectivity matrices. Each of the *N* x *N* entries defines the presence (if >0) and strength of the connectivity between the corresponding source (row) and target (column) region. Standard human functional and structural connectomes are not directed and hence these matrices are symmetrical. **d** Network representation of inter-areal connectivity (grey lines) between nodes (red = hubs; grey = non-hubs). **e** Graph-theoretical measures of segregation (clustering coefficient = blue) and integration (characteristic path length = green). Adapted with permission from Ref. [49]. **f** Complex brain networks (middle panel) combine the segregated properties of highly clustering of lattice-like graphs (left), with the integration of highly-integrated random networks (right). Colours denote community membership of complex, modular networks

Connectomics rests upon a branch of mathematics known as graph theory whereby complex systems are represented as networks of elements (nodes) and their interactions (edges) [44]. Since the seminal “small world” paper by Watts and Strogatz [45], graph theory has grown to influence all of the natural sciences, perhaps nowhere more so than the neurosciences [25], where it forms the basis of connectomics (Fig. 1, Box 1) [26, 31].

The notion of a brain “node”, as a functionally and structurally distinct and homogenous entity depends upon clustering contiguous neural tissue into discrete parcels [46, 47]. The edges linking nodes can then be inferred by applying a suitable measure of connectivity to empirical data (Fig. 1a-c). Research in connectomics divides into structural, functional or effective connectivity, each of which refers to a distinct type of connection or interaction [48]—derived from diffusion MRI (dMRI), fMRI and other neurophysiological recordings. Graph theoretical tools can be used to quantify the resulting (structural, functional or effective) network’s topological organization (Fig. 1d). [49] Network properties can be broadly cleaved into those speaking to segregation or to integration (Fig. 1e) [36, 50, 51]. Of the former, application of the clustering coefficient has revealed highly-clustered patterns of local connectivity in mammalian brains [52, 53]. Such clustering also appears at a more composite scale, segregating communities of nodes into modular-brain structures [54], which may facilitate efficient processing amongst functionally-specialized brain regions [55]. The canonical example of network integration is a short characteristic path length that has been proposed to integrate disparate brain-regions into a highly-efficient system, supporting the functional integration of segregated areas [50]. The co-existence of these complementary network features (integration and segregation) constitutes a “small-world” architecture, a property that the human cortex appears to possess (Fig. 1f) [27, 53].

Many biological networks, including the human connectome, contain highly-connected hubs (Fig. 1d, red circles) [56–58]. Hubs can be simply defined as those nodes which are most strongly connected to the rest of the network, but can also draw from other node-level metrics, including their topological role in global integration (i.e. high betweenness centrality) or inter-module integration (i.e. high participation coefficient) [49, 56, 58, 59]. Structural hubs in the human brain exist predominately within the default-mode network (DMN), particularly it’s parietal and medial prefrontal regions, as well as subcortical regions and the lateral prefrontal cortex (PFC) [26, 56, 58]. Notably, connectivity amongst the brain’s network hubs is enriched, forming a dense anatomical backbone—the rich-club [59, 60]. Despite their high-cost (in regards to their metabolic load and wiring), the rich-club connections of the human connectome integrate disparate “feeder” communities into a global workspace, and thus appear crucial for large-scale, functional integration [56].

If the balance between functional segregation and integration supports adaptive cognitive function, then it follows that any imbalance may lead to cognitive dysfunction and illness expression [50]. While the simple “first order” application of connectomics (connectivity strength) allows for the discovery of over- or under-connected regions in mental health disorders [61], the use of “second order” (graph theoretical) metrics brings a more nuanced picture [38]. In this vein, the notion of SCZ as a dysconnection syndrome was finessed to that of a small world disorder, characterised by a mixture of altered integration (i.e. changes in network efficiency) and segregation (i.e. changes in clustering) [62–66]. These changes have been proposed to underline the characteristic disorganised thinking and perceptual irregularities in SCZ [39, 67, 68]. Recent research has additionally highlighted the impoverishment of the rich-club in those with the disorder [69] as well as those at high genetic risk [70, 71]. These findings speak to the core and persistent cognitive dysfunction at the centre of the SCZ phenotype [72]. Comparable functional disturbances have also been reported in Alzheimer’s disease [42, 73, 74], leading to the framing of both dementia and SCZ as “hub-opathies” of the connectome [75]. Epilepsy has also been positioned as a brain network disorder, with increases in connectivity and segregation centring upon the primary epileptogenic zone [76], and the initiation of generalised seizures facilitated by the rich-club [77, 78].

## Connectomics of bipolar disorder

SCZ, dementia and epilepsy have been the main focus of brain network research and serve as instructive, canonical brain network disorders [29, 38]. More recently, brain network methods have emerged as a frontier in the study of BD. In this section, we briefly revisit traditional neuroimaging investigations of BD, i.e. those that derive from (non-connectomic) studies of abnormal functional activation and morphological changes in patients. We then review the extant literature of structural, functional and effective connectivity alterations in BD to see how these latter studies of integrative processes complement the traditional focus on functional specialization.

### Traditional neurobiological findings in bipolar disorder

**Fig. 2:**
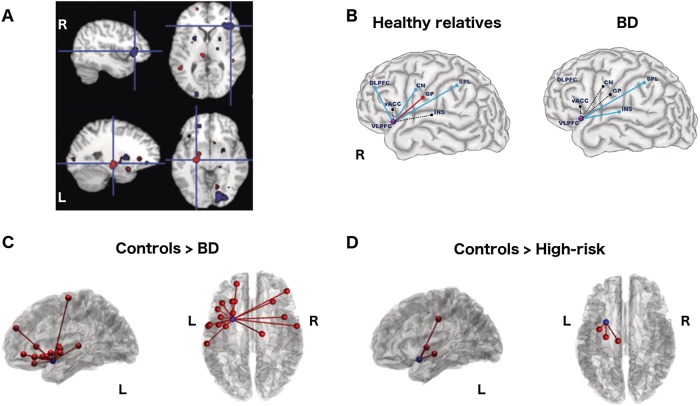
Functional connectivity in bipolar and high risk cohorts: **a** Meta-analysis of functional activation studies in BD shows consistently decreased activation in the right IFG during emotionally salient tasks (blue). Left medial temporal areas show over-activation (red) [21]. **b** Functional connectivity patterns of the right VLPFC (a component of the IFG) during a cognitive task [112], as revealed through psychophysiological analysis. For BD patients (right-panel) and their HR relatives (left), dashed lines indicate reduced task-based functional connectivity patterns. Reduced resting state functional connectivity of the left IFG in BD (**c**) and HR groups (**d**) [111]. The seed node (blue) corresponds to the region with reduced activation in an emotional go-nogo task for HR individuals [117]. vACC, ventral anterior cingulate cortex; CN, caudate nucleus; INS, insula; GP, globus pallidus; SPL, superior parietal lobule; VLPFC, ventrolateral PFC, a component of the IFG

As previewed above, contemporary connectomic conceptualizations of BD build upon earlier studies showing localised morphological and functional disturbances using sMRI and fMRI [13, 21]. One of the most consistent of such findings is increased activity in limbic structures (i.e. amygdala, hippocampus, insular cortex) in BD during emotional processing (Fig. 2a) [16, 21]. The amygdala has traditionally played a central role in models of emotion regulation in the brain, based upon its role in the appraisal of threatening and other emotionally-salient stimuli [14, 79–82]. However, responses to emotional stimuli are also dependent on top-down neural systems involved in the regulation of affect, typified by regions of the PFC [14, 83, 84]. As well documented, the PFC supports a diversity of cognitive-control and executive functions [85–89]. Functional under-activation in the dorsolateral, ventrolateral, ventromedial, inferior frontal and subgenual PFC, during both emotional and cognitive control have indeed been documented in BD (Fig. 2a) [15, 21, 90–92]. In contrast, increased activation has been reported within the ACC [13, 16], although this may be task and mood-dependent, with under-activation also observed during cognitive-control tasks in euthymic patients [93]. Abnormal patterns of activation have also been observed in subcortical and reward-structures including the ventral striatum and the basal ganglia, although the directions of findings are inconsistent and likely context-dependent [16, 18, 128]. Altered reward-based activity is also found in BD patients within prefrontal regions such as the ventrolateral and inferior frontal cortices [94, 95]. The complex interaction between emotional and reward-processing in both health and mood disorders  are currently a topic of considerable interest [96, 97].

Structural neuroimaging investigations focussing on volumetric changes (i.e. sMRI) further support the presence of disturbances in prefrontal, limbic and subcortical areas in BD [98]. For example, structural changes in the amygdala were reported early [99] and consistently [15] in the literature. In brain regions where abnormal activation patterns have been reported, such as the ACC, morphological reductions have also typically been observed [15–17, 90]. A smaller volumetric size of the corpus callosum has also been reported in BD [100–102]. The documentation of structural abnormalities in areas involved in emotional and reward-processing continues to accumulate, with harmonized consortiums yielding very-large cohort comparisons (i.e. thousands of BD patients) [17, 19]. These consortia provide sufficient power to detect subtle reductions in morphological properties of subcortical and cortical areas in BD, as well as the potential neurotrophic effects of lithium [17].

### Functional connectivity in bipolar disorder

In the last decade, a substantial body of connectivity research in BD has built upon these earlier findings, largely by studying connectivity disturbances between regions previously identified as showing local functional and morphological changes [15, 103]. Differences in resting-state functional connectivity have been the most frequently reported. Prominent among these are reports of weaker functional connectivity between the amygdala and regions in the PFC, including the ventrolateral [104, 105], dorsolateral [106], orbitofrontal [107], and medial PFC [108, 109], the IFG [107] and the pregenual ACC [110]. Although the amygdala appears frequently in functional-connectivity studies (often because of its *a* priori selection as a region of interest), reduced functional connectivity in BD has also been observed to involve other regions—between the IFG and the ACC (Fig. 2b) [111, 112], amongst medial prefrontal areas [113], as well as a distributed pattern of connectivity between the medial nucleus of the thalamus and several disparate regions of the cortex [114]. Functional connectivity disturbances in reward-circuitry during resting state and reward-based tasks have also been observed [15, 115, 116], most notably involving the ventral striatum, with decreased connectivity strength co-varying with depression severity in patients with bipolar (and unipolar) depression [115].

While these studies provide insights into the mechanisms underlying emotional dysregulation in BD, they are effectively constrained to local patterns of network dysfunction, typically limited to seed-based analyses or specific pair-wise interactions [103]. Moving toward network analyses in a multivariate framework can be achieved using Network based statistics (NBS), a technique that exploits the topological properties of interconnected subnetworks of edges to control for family-wise error [61]. NBS analysis of BD patients has been applied to study functional connectivity of the ventromedial IFG, [111] a region showing decreased engagement for HR (i.e. high risk) individuals in an emotionally-salient cognitive control task [117]. Functional dysconnectivity of this area in BD patients involves a constellation of fronto-limbic-striatal regions, including the bilateral insular cortex, ventrolateral PFC, superior temporal gyri, and also the putamen (Fig. 2c). Multivariate functional connectivity can also be studied using independent components analysis (ICA), which aggregates regions into maps, or resting-state networks (RSN’s), based on correlated patterns of intrinsic fluctuations [118]. The application of ICA to BD has revealed alterations within and between RSN’s containing fronto-limbic, default-mode, thalamic, cognitive-control and somatosensory regions [119–123]. Differences have also been observed in the default mode (DMN) [124–126]—a constellation of regions that are less active during external task execution [127].

As discussed above, graph theoretical tools allow the topological organization of brain networks to be interrogated. Despite the paucity of studies, the effects on whole-brain intrinsic functional organisation in BD are equivocal or negligible: weak effects reported in the characteristic path length do not survive correction when considering the five other network metrics that were also analysed [128]: this accords with the null findings reported in two well-powered studies [111, 129]. Regional topological effects (in subnetworks focussed on specific regions) have been observed, with changes in both the topological segregation and/or integration of areas. These regional-level functional connectivity changes in BD patients correspond to decreased segregation (decreased clustering coefficient) of the ventromedial IFG [111], and opposing patterns of both increased and decreased integration in the DMN, sensorimotor cortex, occipital areas, cerebellum, temporal pole, mid-cingulate cortex and the dorsomedial PFC [128, 129]. However, a more definitive interpretation of the topological changes in BD are currently limited by the small number of graph-theoretical functional connectivity investigations, and the different graph metrics used across studies. Nonetheless, on available evidence it seems reasonable to conclude that in contrast to the strong distributed effects in SCZ, the functional network topology in BD appears to be confined to specific functional subsystems.

### Structural connectivity in bipolar disorder

The properties of structural brain networks can be inferred from dMRI data using a variety of approaches. These can be divided into estimates of local “white matter integrity” using voxel-wise measures of diffusion such as fractional anisotropy (FA) and mean diffusivity (MD), or by applying tractographic techniques to reconstruct whole-brain structural networks and then subjecting these to graph theoretical analyses (Fig. 1). Of the former approaches, FA and MD have been most extensively employed, using either a voxel-based approach (similar to the analysis of regional morphological changes in sMRI) or tract-based spatial statistics (TBSS) which, as the name suggests, allows analyses of FA along “skeletonized” white matter tracts. Changes in FA have been consistently demonstrated within the corpus callosum [130], particularly the anterior horn (connecting bilateral prefrontal and limbic regions) [131–134]. The application of TBSS to BD has revealed decreases of FA in intra-hemispheric white matter bundles interconnecting pre-frontal, limbic, and subcortical structures, such as the cingulum bundle [133, 134], uncinate fasciculus [135, 136], the anterior thalamic radiation [135], and the superior longitudinal fasciculus [130].

Substantial “DTI-based” research has thus contributed to the notion of structural dysconnectivity amongst limbic and prefrontal regions in BD [132]. However, interpreting the biological underpinning of FA (and other DTI-based metrics) is problematic [137, 138]: the diffusion signal itself reflects a number of underlying contributions, including the relative proportion of white and grey matter, the degree of axonal myelination, the presence of crossing-fibres, and the presence of extracellular changes leading to free water [139]. Despite extensive use to the contrary, DTI-derived metrics such as FA should hence not be interpreted as directly reflecting the “integrity” of white-matter connectivity [137, 140]. While differences of FA in BD may reflect microstructural changes, they are likely also confounded by white matter volume, connectivity geometry (e.g. crossing fibres) and, possibly, free water from neuroinflammation. A multi-modal approach employing susceptibility imaging, free water imaging and quantitative mapping is required.

**Fig. 3:**
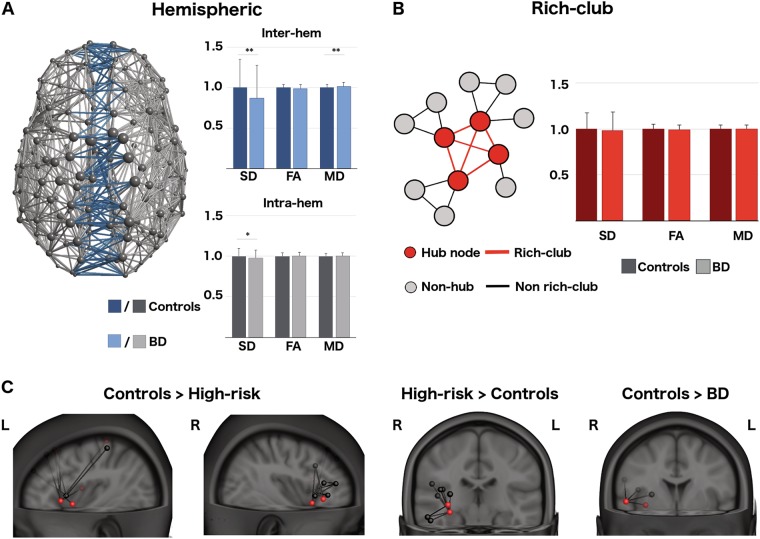
Structural connectivity in bipolar and high risk cohorts: **a** Structural brain network comprised of intra-hemispheric (grey) and inter-hemispheric (blue) connections. Inter-hemispheric connections indexed by streamline density (SD) are weaker in BD compared to controls [101]. Connectivity estimates using FA or mean diffusivity (MD) do not show group differences. **b** The structural rich-club corresponds to the clique of highly connected hubs that have an enriched hub-to-hub connectivity. There are no apparent group differences in rich-club connectivity in BD, whether using SD, MD or FA [101]. **c** Group-wise structural connectivity subnetworks in BD, HR and control groups revealed using NBS [141]. FA, fractional anisotropy; SD, streamline density; MD, mean diffusivity

Rather than focusing on local white matter properties, tractography performed upon dMRI data allows examination of large-scale brain networks, with the “connectivity” of these networks typically corresponding to the streamline density. Mirroring the morphological disturbances in the corpus callosum, analyses of tractography also supports a disturbance to the integration of inter-hemispheric structural connectivity (Fig. 3a) [100, 101]. The application of NBS to whole-brain tractography, allowing a focus on specific subnetworks, reveals diminished connectivity amongst a small subnetwork centered on the right rolandic operculum—a region bounding the insula and IFG—and extending into medial temporal regions (Fig. 3c) [141].

As with functional connectivity, graph metrics can also be applied to tractography both at the whole-brain (to reveal large-scale topological disturbances) and nodal-level (to examine the network environment of specific brain regions). Such an approach has revealed local structural network changes largely in prefrontal and limbic areas, namely, the hippocampus, IFG, anterior insula (AI) and superior frontal gyrus [132, 141]. These findings include reductions in connectivity strength (i.e. nodal strength) [141] and local decreases in both integration (reduced efficiency and increased path length) and segregation (decreased clustering) [100, 142]. Interestingly, reports of group-wise differences in node-wise structural connectivity have not identified the amygdala, regardless of whether whole-brain or a priori analyses have been conducted. However, the amygdala is a deep nucleus with a relatively small volume, hence inter-subject variability in tractography estimates may require more innovative means of accounting for anatomical variability than used in standard analyses.

Application of graph-theoretical tools to whole-brain networks has yielded a more nuanced picture: slight decreases in network integration have been observed in BD (using either the characteristic path length [141] or the comparable global efficiency [101]), although the latter did not survive family-wise corrected threshold after controlling for group differences in IQ. Differences in whole-brain segregation, using the clustering coefficient, have also been reported, although in contradicting directions (i.e. both lower [100, 143] and higher [141]), possibly due to differences in dMRI acquisition and analysis [144]. Notably, several well-powered studies have suggested preserved integrity of the rich-club in BD (Fig. 3b) [101, 141]. Two slightly smaller studies reported marginally significant changes in the rich-club connectivity of hubs [143] or their connections [145]: however, these effects do not survive appropriate multiple-comparisons correction. This is in opposition to the pervasive disruption to the structural rich-club that is characteristic within SCZ [39], suggesting that changes in the core anatomical backbone may be a potential marker that discriminates between the two disorders.

In sum, disturbances in large-scale functional and structural networks in BD appear subtle and are more likely to be confined to specific regions and subnetworks in limbic and prefrontal regions. The subtle decreases in whole-brain integration observed in BD may reflect alterations to inter-hemispheric connectivity [101], as well as the disrupted connectivity of prefrontal-limbic circuits. When benchmarked against the widespread disturbances in SCZ [62, 63, 66–70], it is evident in BD that most of the structural and functional “backbone” of the connectome is left intact. This is consistent with the cardinal differences in the phenotypes, specifically the relative inter-episode preservation of cognition and affect in BD [146]. Future network-based research is required to better characterise the preferential disruption of local rather than the large-scale-connectivity in BD. In particular, it remains unclear whether the subtle whole-brain effects that are seen do indeed reflect a pernicious, whole brain change in network topology or are rather local network changes that are sufficiently severe to be reflected in whole brain measures (which are composite averages across all nodes).

### Effective connectivity in bipolar disorder

Structural and functional connectivity thus represent novel candidate diagnostic markers for the non-invasive identification of BD. They can be acquired with minimal imposts of time and training for patients. However, when it comes to understanding network mechanisms, these advantages are somewhat of an Achilles heel, as linking the observed network disturbances to the BD phenotype in the absence of a task rests largely upon correlational analyses, that is, of regressing connectivity changes against contemporaneous phenotypic variables. Functional interpretations are also prone to reverse inference [147], namely imputing that a disturbance in a neural substrate (structure, circuit or network) underlies the affective or cognitive disturbances of BD because that substrate is engaged by a specific task in a different context. This logic rests upon a flawed assumption of a one-to-one structure-function coupling [36], and fails to recognise the cross-modal and functional multiplicity of many cortical regions. Notably, most of those regions that frequently figure in connectomic studies of BD, including the AI, IFG, ACC and dorsolateral PFC (DLPFC), are precisely those that are most frequently engaged across a broad variety of cognitive and emotional tasks [148]. So, although their involvement in connectomic studies supports the notion of dysregulation of emotional networks, the inference is indirect and other interpretations are possible.

Task-related effective connectivity studies largely eschew these problems because they interrogate network disturbances in clinical populations during performance of specific tasks. The most widely used technique for studying effective connectivity is dynamic causal modelling (DCM) [149] which uses Bayesian inference to identify the network model of effective connectivity most likely to have generated observed task-fMRI data [150]. To date, there have only been a handful of papers employing DCM to study the connectomics of BD and most of these have examined effective connectivity during perception of facial affect. For example, BD is associated with decreased effective connectivity between the DLPFC and the amygdala during the during perception of angry and fearful faces [151]. During perception of happy faces, decreased effective connectivity from the orbitomedial PFC to the amygdala has been observed in patients with bipolar, but not unipolar, depression [152]. In BD, disambiguation of emotionally expressive faces from those with a neutral expression is associated with weaker effective connectivity from the amygdala to the ventral PFC [153]. Intriguingly, clinical response to chronotherapy (sleep deprivation combined with light therapy) is associated with increased effective connectivity from the DLPFC to ACC in those with BD depression [154].

While few in number, these studies of effective connectivity are important steps toward identifying the neural mechanisms of phenotype and treatment response in cognitive and emotional networks in BD. They demonstrate, for example, that interactions amongst key brain regions (amygdala, DLPFC, ACC, ventrolateral PFC [VLPFC]) are indeed disturbed during the perception of facial affect.

## Connectomic investigations of high-risk cohorts

Several limitations beset studies of BD: the confounding effect of different classes of medication (mood stabilisers, antidepressants, antipsychotics), comorbidity, and the possible secondary effects of illness expression. Waiting for the expression of a manic episode to confer the diagnosis also misses the opportunity to mitigate the secondary harm (e.g. to reputation, and risk of suicide) if the illness could be averted. Addressing these important issues can be achieved through the study of unaffected HR cohorts. BD has a strong familial association and hence offers the potential for disambiguating risk endophenotypes [155–157] and temperamental variations [158] from markers of illness-expression through the study of HR individuals [155, 159], such as first-degree relatives of patients with BD. The peak age for BD illness onset is within the early twenties [7]: HR individuals within this age-range are thus amongst the highest risk: understanding conversion to illness in this age bracket must be disambiguated from the complex maturational processes of late adolescence and early adulthood [160–162]. Studying those HR individuals who do not develop BD, despite a higher background risk, offers the equally important opportunity to study factors underlying illness resilience [129]. Despite the relatively large literature on traditional morphological and functional activation studies in HR cohorts [163], there exist relatively few HR connectomic studies.

### Structural connectivity in high-risk cohorts

A recent study of structural connectivity compared a young HR cohort (mean age 22 years; range 15–30) to matched control and BD cohorts [141]. Application of NBS identified two lateralized subnetworks weaker in the HR group, based upon reductions in streamline density; each network centred on a number of structural hubs including the AI and ventro-lateral IFG. These networks involved connections with the posterior insula, medial PFC, superior temporal gyri, somatomotor cortices, and the ventral-striatum (Fig. 3c) - regions associated with cognitive, emotional and somatosensory functions. Despite involvement of rich-club hubs in these subnetworks of weaker connectivity, the connectivity of the rich-club itself was not disturbed in the HR group in this, or an independent study [71, 141]. That is, hub-to-hub connections appear to be preserved. Intriguingly, a subnetwork of increased connectivity, centered on the right hippocampus, was also present in the HR cohort. Also of note, these HR subnetworks were not disrupted in the matched BD group, who instead expressed their own subnetwork of weaker structural connectivity (Fig. 3c). Alterations that were common across BD and HR groups included the node-wise connectivity of superior frontal, hippocampal, and mid-occipital areas.

Older HR individuals, who have passed the peak age for BD onset, also represent a unique study population. Whereas young HR groups are a mix of risk and resilience, older HR cohorts more likely express neurobiological patterns that reflect resilience [22, 23]. A structural connectomic investigation of a HR cohort involving older individuals (average age 43; range 21–64), did not identify any group differences. This contrasts with the relatively strong effects seen in young HR individuals. However, this null finding may be attributable to the weaker MRI field strength (1.5T) and the use of simpler fibre-reconstruction methods that do not reconcile crossing-fibres.

The majority of structural connectivity studies in BD and HR cohorts have been derived from streamline-based approaches. As with the earlier use of FA, the extent to which streamline counts correspond with the intra-axonal “fibre density” is debatable. Recent streamline-filtering approaches provide more biologically accurate measurements [164], but require innovative acquisition sequences and their uptake into clinical studies has hence been slow. Acquisition and modelling techniques have been recently developed which delineate specific microstructural properties, such as neurite density [165] and extracellular content measures [166]. Future connectomic studies of BD and HR populations are recommended to leverage these techniques to reconcile microstructural abnormalities with large-scale network changes.

### Functional connectivity in high-risk cohorts

As with structural connectivity in BD and HR cohorts, the large-scale network topology of resting-state functional connectivity in HR cohorts also appears to be largely conserved [111, 129].

Reported disturbances are again confined to specific subsystems of the brain: increased functional integration (i.e. increased participation index) has been observed within default-mode regions in an older HR cohort (mean age 32) but not matched control or BD individuals [129]. In the same study, both BD patients and their unaffected HR siblings showed increased functional integration in regions of the sensorimotor network and higher-order visual regions. No other study has investigated whole-brain functional topological changes in HR individuals.

Task-related functional connectivity has been probed in HR cohorts using psychophysiological interactions (PPI) [167], a method that identifies changes in functional connectivity between two regions coincident with a cognitive manipulation. During a linguistic stroop test, VLPFC connectivity with the vACC was reduced in both BD and older HR participants (average age 36) (Fig. 2b) [112]. In the same study, VLPFC connectivity with the insula was also affected: reduced VLPFC–insula coupling was uniquely present in HR group compared to controls, whereas BD patients showed a change of sign in this PPI association from negative to positive.

Resting-state functional connectivity can also be used to examine whether task-related effects persist beyond the context in which they were observed. In this vein, the left ventromedial IFG, whose engagement in an emotional ‘go-nogo’ task was reduced in young HR individuals (average age 23 years) [117], also showed a distinct patterns of reduced resting-state functional connectivity in BD and HR cohorts; in HR, functional connectivity with the neighbouring AI and the ACC was decreased (Fig. 2d) [111]. The pattern of decreased functional connectivity in BD compared to controls is quite distinct. Application of machine learning techniques to these data identified a distributed, bilateral subnetwork of weaker resting-state functional connectivity which provided reasonably accurate three-way classification of participants into their groups (control, HR and BD; 64% accuracy benchmarked against a 41% chance rate).

### Effective connectivity in high-risk cohorts

**Fig. 4:**
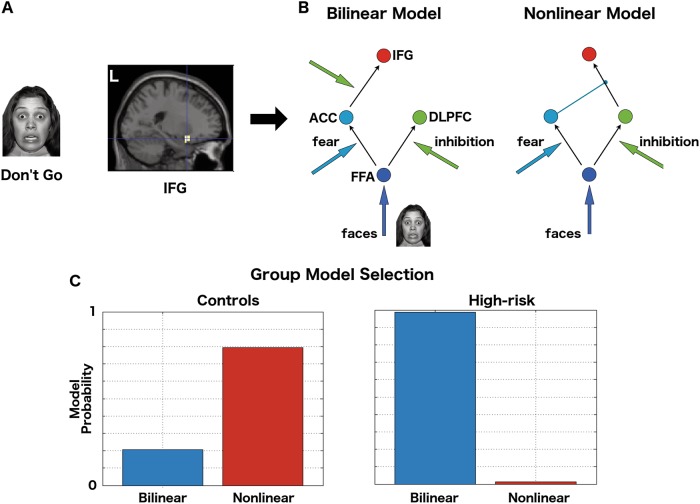
Effective connectivity in high risk cohorts: **a** Hypo-activation of the left IFG in HR individuals when inhibiting a motor response to a fearful stimuli (from an affective Go-Nogo task) [117]. **b** The network dynamics generating these data were then modelled using DCM [168]. Models consisted of inputs (faces) delivered to the FFA, with effective connections to the DLPFC modulated by motor inhibition to explain the effect of the “NoGo” trials. Effective connectivity of the FFA to the ACC was modulated by the presence of fear faces. Bilinear models then added an additional modulatory influence (here, inhibition) to explain the interaction between motor inhibition and fear on the IFG. Nonlinear models introduced this interaction through internal gating effects (here the influence of the ACC on the effective connection from the DLPFC to the IFG. **c** Model (exceedance) probabilities using Bayes model selection. For controls, the nonlinear models were the most likely family, that is, those models with nonlinear (gating) effects of the ACC on the DLPFC to IFG connectivity. In the HR group, the corresponding nonlinear parameter showed a between group difference and the most probable family of models were the bilinear ones, that is, those models where the internal nonlinear effect has been replaced by an external modulatory influence. ACC, anterior cingulate cortex; DLPFC, dorsolateral prefrontal cortex; FFA, fusiform face area; IFG, inferior frontal gyrus

The left IFG, particularly the ventro-lateral and ventro-medial portions, have thus been implicated in task-related hypo-activation as well as reduced functional and structural connectivity in both young and older HR individuals (Figs. 2a–c, 3c). The presence of reduced functional and structural connectivity suggests an intrinsic network disturbance that may underlie the task effect. Nonlinear DCM, which permits analyses of complex network effects during task execution was recently employed to probe the mechanisms underlying this hypo-activation pattern observed in HR individuals (Fig. 4) [168]. In controls and those with BD, a hierarchical network of interactions between the ACC, the DLPFC and the IFG provided the most likely explanation of these data: this network motif allows a balanced convergence of cognitive control and emotional salience on the IFG. In the young HR group (a subset of those where reduced structural [141] and functional connectivity [111] was reported), the hierarchical gating of the ACC on the effective connectivity between the DLPFC and the IFG was diminished. Intriguingly, the most likely network model of effective connectivity in the HR group was distinct from both the control and BD groups.

A DCM study of effective connectivity in an older HR cohort (BD siblings, mean age 39.7) reported significantly increased effective connectivity between the inferior occipital cortex and the fusiform gyrus during perception of emotionally salient faces [153]. This increase in effective connectivity was not found in BD patients. BD patients, in common with HR relatives, demonstrated increased effective connectivity between the amygdala and right VLPFC, highlighting fronto-limbic interactions as a marker of illness risk and expression for BD.

Hence, there are patterns of structural, functional and effective connectivity that are unique to HR cohorts when compared to BD, as well as shared patterns. Disturbances of the connectivity and functional involvement of IFG in broader prefrontal-limbic circuitry appear to be more prominent in those at risk than those with the disorder. Functional under-activation of the IFG and the surrounding VLPFC during emotional and cognitive control are common to both HR and BD populations, as are distributed disturbances such as in somatosensory and higher visual cortex.

Integrating the connectomic findings in HR individuals is challenging, given the small number of studies that exist and their cross-sectional nature. In addition, these studies have been conducted on HR individuals at different developmental stages of risk for BD. As we argued above, young HR individuals are at a critical cognitive and affective developmental period. Disturbances to the IFG and its affective and cognitive connectivity in young HR cohorts may reflect an endogenous risk that changes upon illness expression in those whom convert to BD. Alternatively, unique differences in older HR individuals may also represent early compensatory responses that confer resilience [112, 168, 169] in those whom do not develop manic episodes. The following sections consider the potential for elucidating disease mechanisms in longitudinal investigations of HR populations.

## Challenges and opportunities in BD connectomics

In sum, viewing BD through the lens of connectomics positions earlier studies of functionally localised disturbances into broader circuit and network mechanisms that integrate cognitive control, affective and reward-systems of the brain. Some connectivity disturbances are common to both BD and HR groups; these speak to a shared risk of affective dysregulation. Other connectivity disturbances are unique to BD, reflecting illness expression and possible medication effects. Several changes are unique to HR cohorts: in the younger of these, unique effects may reflect a mix of risk and resilience, whereas resilience may figure more highly in older HR groups. When interpreting these effects, however, it should be recalled that the actual inherited risk of a first-degree relative is far from uniform and varies sporadically and with the severity of the affected relative (e.g. a higher risk is associated with an early age of onset of BD). Thus, unaffected older first-degree relatives may have a lighter genetic load, as reflected in the absence of structural connectivity differences [142] or a stronger adaptive response to the same genetic load as their BD relative. Genetic risk also interacts with early life experience to impact on illness expression [170]. Disambiguating these scenarios cannot be achieved with cross-sectional imaging studies but require longitudinal studies informed by assays of early life stress and genetic risk markers such as the BP polygene risk score.

### The potential of longitudinal and genetic-risk studies

The first expression of BD typically occurs during the final neurodevelopmental stages of brain development and co-occurs with fundamental changes in inter-personal functioning, role, and self-identity [171]. Longitudinal HR studies, sufficiently powered to enable multivariate analyses of connectomic, phenotypic and genetic data are required here. Such analyses should also accommodate the complex developmental trajectory of brain networks [172, 173]. The connectomic correlates of current mood states and current and lifetime episodes are largely unknown. The few direct comparisons of BD mood states and subtypes do suggest the existence of both trait and state network markers [174–176]. However cross-sectional studies of remitted versus in-episode BD patients cannot address within-subject variations in phenotype. Longitudinal designs are particularly pertinent for young high-risk BD groups who later develop sub-threshold or threshold BD symptoms. Existing (non-connectomic) studies have reported different baseline fMRI activations to cognitive [177] and emotional-tasks [178, 179] in those HR individuals who later develop a first depressive episode. Repeat functional imaging assays also suggest different trajectories of functional activation in the striatum and insula of unipolar versus bipolar depression [180]: morphological studies using sMRI likewise suggest predictors of first episode mood disorders in HR populations, [181, 182] as well as the likelihood of further mania in those with BD [183]. Moreover, different trajectories of volumetric development have also been observed in HR individuals who developed major depressive disorder relative to those who remained well [181]. Intriguingly, while fractional anisotropy (FA) in a HR cohort differed from controls at baseline, corresponding changes in FA at follow-up did not differ [184].

Although the potential utility of brain network analyses to identify future psychosis [185] in high-risk psychosis cohorts has been established, longitudinal investigation of high-risk BD populations are yet to be interrogated with such tools.

Longitudinal studies which integrate imaging assays with genetic risk variants for BD may also help disentangle aetiological mechanisms. Patient carriers of genetic risk variants for BD (CACNA1C and ANK3) show abnormal effective connectivity in facial processing networks [153]. Potential for elucidating the genetic pathways underlying BD may also lie in leveraging an individual’s cumulative load of candidate genes for the disorder, known as a polygenic risk score (PRS) [186]. In recent studies conducted in BD and family-relatives, PRS was found to be associated with functional brain activity in working memory and facial processing networks [186, 187]. Intriguingly, the loading of PRS on brain activity was independent of diagnosis. Understanding the complex interaction between imaging markers, genetic risks and illness expression should be an important goal of future longitudinal studies.

### The need for multi-disorder studies

Several clear observations emerge from the growing body of BD connectomics research. The over-arching picture is that, compared to the pernicious “small world” and rich-club effects in SCZ, disturbances in BD and HR populations appear to be confined to specific fronto-limbic subsystems, most notably those associated with the perception and regulation of emotionally salient material. Whereas large consortia investigations have revealed that all major white-matter bundles are impacted to some extent in SCZ [166, 188], the emerging consensus of the work we have reviewed suggests a more restricted pattern of connectomic disturbances in BD. This difference is even apparent in unaffected offspring—those of SCZ patients show a decrease in rich-club connectivity that is not present in the offspring of BD patients [71]. The differing connectomic signatures between BD and SCZ are interesting in light of the substantial genetic overlap between the two disorders [189], and the shared cognitive deficits in executive control [146]. Interestingly, a common pattern of decreased resting-state functional connectivity in the frontoparietal control network has been observed across SCZ, schizo-affective and BD [121, 190].

As noted above, these connectivity differences reflect the over-arching distinction of the phenotypes: the bivalent and episodic nature of BD contrasts with the core and enduring perceptual and cognitive deficits that characterise SCZ. Hence, even though the networks of structural, functional and effective dysconnectivity in BD do “hang off” important brain hubs such as the AI, IFG, DLPFC and ACC, the connectivity amongst other distributed core-regions remains relatively preserved. However, the degree of disturbance in those networks that are affected in BD is quite substantial. That is, the disorders may differ in their core topological reach, but not in the depth of the perturbation on the networks where they do impact.

Unfortunately, most prior connectomic studies of BD and SCZ patient groups are typically careful to exclude each other, clinical practise suggests a less distinct continuum of phenotypes that reflects the shared genetic architecture [157]. Given that the differential diagnosis of SCZ from BD is a frequent clinical challenge, further large cross-disorder studies are required [126].

On the topic of differential diagnoses, the need to detect BD during its initial depressive episodes is crucial to early and targeted interventions. The potential utility of neuroimaging markers to differentiate bipolar from unipolar depression has been highlighted by the few connectomic studies that include both BD and unipolar (UD) depression cohorts [128, 152, 176, 191]. Disruptions to resting and effective connectivity patterns (during facial processing) between the amygdala and medial PFC have emerged as unique to BD [110, 152]. Alterations to reward-based circuitry have also emerged as a potential marker to discriminate BD from MDD patients [115]. To date, the only graph-theoretical comparison of BD (in this case, type II BD) and UD patients revealed patterns of both shared and unique resting-state functional-network abnormalities [128]. Shared topological alterations occurred in fronto-limbic areas, whereas increased nodal integration of the precuneus was found for UD patients, relative to BD. As with SCZ, cross-disorder studies are required if imaging is to translate into clinical practise. To achieve this, connectomic studies, as with earlier functional activation studies, require carefully chosen cognitive and emotional probes, as reflected in the valence-dependent nature of amygdala activity in BD versus UD [128, 152]. Naturalistic stimuli, such as emotionally salient film and news clips - have been used to identify functional [192, 193] and effective [194, 195] connectivity correlates of UD subtypes and might also play a role here. In common with resting-state acquisitions, dynamic natural stimuli can be easily translated into clinical populations, while also incorporating specifically timed cognitive and emotional material [192].

In addition to multi-disorder studies, successful clinical translation rests upon replication in large, independent studies [196]. This approach tests generalizability across the nuances of site-specific patient recruitment and scanner imaging quality. Independent studies that test prior (published) effects also offer protection against the “researcher degrees of freedom” that characterise much historical discovery research [197]; larger studies improve the accuracy of the estimated effect size [198]. Data sharing platforms such as ENIGMA can play a crucial role here [17]. However, the influence of large legacy data sets on connectomics may be limited by the need for advanced imaging sequences, particularly the need for high-angular and distortion-corrected diffusion images to improve the accuracy and reduce the biases of tractography [144]. Testing task-related effective connectivity also requires harmonization of task design and that can often only be achieved prospectively.

### Fusion of multimodal neuroimaging data

**Fig. 5:**
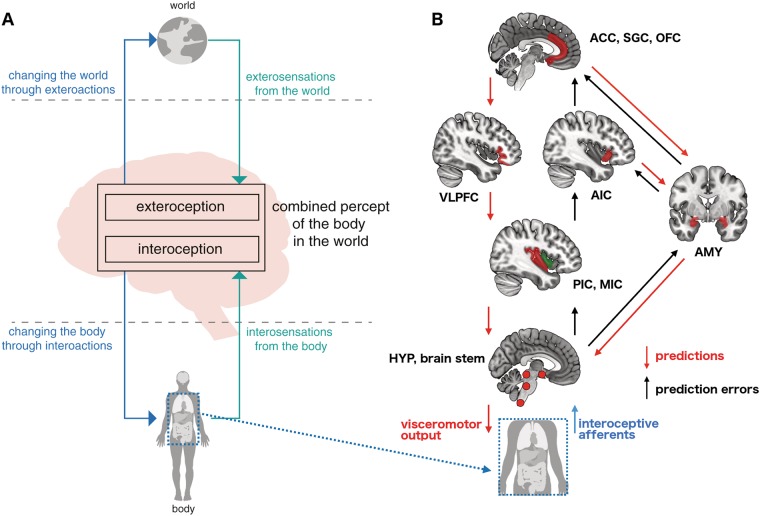
Computational models of interoception and mood: **a** Classic perception (“exteroception”) involves an action-perception cycle, acting through motor behaviour (e.g. eye or limb movement) and perceiving (or inferring) things in the world through vision, proprioception etc. Interoception involves a similar action-perception cycle with internal systems: Physiological states are primed through efferent visceromotor effects, and likewise sensed by visceral afferents. Adapted with permission from [257]. **b** Hierarchical interoceptive processes involve an action-perception cycle between the central nervous system (CNS) and the body through descending visceromotor efferents and ascending autonomic inputs to the spinal cord, brain stem and hypothalamus (bottom, red circles). These then engage in active perception through the mid-and posterior insula (second from bottom), anterior insula (second from top, right panel), IFG/VLPFC (second from top, left) and cognitive-control regions (top). The amygdala (far right) acts in parallel, activating fear circuits in response to threat-salient prediction errors. At each level, descending arrows represent predictions which are compared to ascending inputs: The mismatch yields a prediction error which is feed up the hierarchy to update internal models (i.e. mood and expectation). ACC, anterior cingulate cortex; AIC, anterior insula cortex; AMY, amygdala; HYP, hypothalamus; MIC, middle insula cortex; OFC, orbitofrontal cortex; PIC, posterior insula cortex; SGC, subgenual anterior cingulate cortex; VLPFC, ventrolateral prefrontal cortex

Computational models of brain activity suggest that the structural connectome forms a scaffold that shapes complex, multiscale neuronal dynamics [35, 199–202]. Accordingly, structural connectivity in healthy adults is a strong predictor of corresponding functional connectivity [203, 204] and can be used to constrain models of effective connectivity [205]. Quantifying structure–function relationships in clinical populations may be instrumental in revealing disorder mechanisms [206]. For example, multimodal analyses could assess whether structural abnormalities in HR populations overlap with subject-wise differences in functional connectivity. As we have seen, several findings in BD connectomics span structural, functional and effective connectivity - changes in the left IFG are an exemplar (Fig. 2–5). Despite this, there are currently very few network-based structure-function investigations in BD or HR cohorts [71, 207, 208]. One study of a young HR cohort in a young HR cohort (mean age 14.2 years) revealed increased correspondence between structural and functional connectivity over long-distance connections [71]. The fusion of connectomics with complex clinical and behavioural data using multivariate statistical approaches [209–211] may also help identify those brain network changes that are most salient to the phenotype.

## Future directions: from networks to dynamics, system control and instabilities

The emergence of computational psychiatry approaches [212] has shifted research from traditional nosological group-based investigations towards individual patient mechanisms and predictions [213, 214]. The mechanisms linking neurobiological disturbances and phenotype are being studied using computational models of perception, learning, inference and behaviour [214, 215]. For example, the involvement of dopaminergic reward circuits in BD [115, 216] has motivated computational accounts of the influence of unstable mood fluctuations on reward-based learning [96, 217] and decision-making [97]. Accordingly, abnormalities in reward-based activity in the left VLPFC has been identified in BD patients [94, 95]. Meanwhile, large-scale biophysical models offer a principled way of understanding how complex, multiscale neuronal dynamics emerge from local neuronal populations interacting through the structural connectome [33–35, 218, 219]. Computational studies also allow in silico manipulation of system dynamics in a manner that cannot be achieved in experimental studies [220]. We offer ways of bringing these computational methods to bear upon our understanding of BD connectomics.

### Network dynamics and system control

Dynamics lie at the heart of BD: On short time scales, fluctuations in arousal and motivation are a core aspect of everyday life [221]. When appropriately bounded, slower changes in mood and affect are also an important component of adaptive interpersonal functioning: they bias learning [217] and prime expectations according to anticipated social context. Computational studies of mood swings in BD can be conceptualized as a failure in the appropriate regulation of these fluctuations: Time series analyses suggest mood variations in BD undergo a switch to “chaotic” dynamics [222]. Although these results need to be considered in light of the caveats of nonlinear time series analyses [223], they suggest an intriguing role for dynamic models of brain activity to disclose the origin of multiscale temporal phenomena. Several of these models have linked topological features of the connectome to the emergence of multiscale neuronal dynamics [35, 218, 224, 225]. Intriguingly, a recent study has shown how the enriched connectivity of cortical hubs leads to the emergence of slow, stable synchronous dynamics in the structural core, in contrast with the fast and unstable fluctuations in the topological periphery [199]. Notably, the slow core encompasses much of the emotional circuitry and cognitive control systems whereas fast, peripheral regions typically lie in primary sensory cortex. A hierarchy of such timescales finds support in a diversity of invasive neurophysiological [226] and functional neuroimaging data [227]. These “chronoarchitecture” findings suggest that the (slow) time scales of the emotional hubs are matched to the slow time scales of internal states (such as changes in mood), whereas peripheral regions are tuned to the fast fluctuations of events in the external sensorium [199]. Corresponding structural disturbances involving these core hub-regions could then destabilize the corresponding slow dynamics in BD.

What network mechanisms might cause this? Insights could derive from the application of network control theory, that is, quantifying how inputs to individual nodes or subnetworks potentially change the state of the whole network [228, 229]. The application of this framework to empirical data predicts that the human connectome is indeed controllable (i.e. can be manipulated to any state), with high degree hubs in the default mode system allowing the transition of brain activity to many diverse states [230]. Controllability of nodes largely arises through their strong connectivity, but also reflects local network topologies such as clustering and local closed paths [231–233]. Recent re-analyses of structural connectivity data shows that the left-sided subnetwork centred on the left IFG that is more weakly connected in HR participants (Fig. 3c), shows reduced controllability in BD patients compared to the control group [233]. The resultant loss of controllability in BD is consistent with the loss of dynamic stability of mood fluctuations in patients and the subtle affective changes in HR individuals.

Currently, such applications are grounded in linear network control theory. While linear approximations may be sufficient to capture state-to-state transitions, future developments in the more elusive nonlinear network control theory may reconcile this approach with the richer dynamics present in empirical data [33, 234].

### Interoceptive models of emotion

It finally remains to be seen how and why the networks implicated in BD play a role in the (dys)regulation of mood. For this purpose, we highlight recent developments in computational psychiatry that reconcile a ‘William Jamesian’ mapping between physiological states and emotion with predictive coding accounts of perception and inference [11, 235, 236].

Emerging frameworks of emotional experience and psychopathology have repositioned brain function in light of its interactions with internal body systems [11, 235]. These interactions are proposed to occur through a perceptual process known as interoception: physiological changes signalled through autonomic and visceral inputs to hypothalamic and brainstem structures are sensed “from within” and yield corresponding neuronal responses in limbic cortices [237, 238]. According to William James’ account of emotion, such sensations (of aroused physiological states) map onto corresponding emotional states (fear, anger, apprehension, etc.). These processes mirror the more familiar exteroceptive systems (vision, hearing, etc.) in terms of their salience and the hierarchical structure of corresponding sensory cortices: whereas visual systems feed-forward and back from the occipital pole, primary and higher-order interoceptive cortex project between the posterior and anterior insula, and also the IFG [11]. As with exteroception, sensory processes in the interoceptive system are primed and contextualized through cognitive control processes such as attention, expectation, and inhibition mediated via prefrontal regions: The state-dependent nature of these contextual processes correspond to mood [236].

Following Bayesian ideas, contemporary models of active perception rest upon hierarchical representations of sensory signals encoded as prediction signals in agranular cortices, namely the AI, ACC, subgenual cortex (SGC), and also the orbitofrontal cortex (Fig. 5b) [11, 239–242]. Descending predictions of (internal and external) states are compared to the ascending sensory input from lower-order regions with the difference between the two serving as a prediction error [243, 244]. The signal encoded in this prediction error actively updates the higher-order representations. Heteromodal regions, such as the IFG, may modulate the magnitude of this update according to the certainty or “precision” of the sensory inputs [245]. In classic (external) perception, this process ensures useful actions and reliable models of the physical world [244]. In interoceptive systems, these processes reconcile physiological states with social context and potential threats (Fig. 5a). This serves homeostatic needs by efficiently allocating physiological resources to appropriate cognitive, emotional and behavioural states whilst minimizing energy expenditure. Prediction errors also serve allostatic processes by engaging visceromotor responses, causing physiological changes that are central to our affective content and feelings, such as heart palpitations and blushing [11, 239]. If a threat is anticipated then autonomic processes are primed accordingly. Large prediction errors create arousal, surprise and re-orienting. Emotional experience can hence be considered a circular process, such that interoceptive signals inform and update these prior beliefs [11].

### Integrating connectomics and computational psychiatry: bipolar disorder as “interoceptive psychosis”

We conclude by considering how this framework can be used to integrate the various connectomic disturbances in BD and HR cohorts. To this end, it is notable that the agranular cortices proposed to embed interoceptive predictions comprise the prefrontal and limbic areas [11, 239, 246] repeatedly implicated in BD. As reviewed above, cognitive and emotional processes are supported by these areas, exemplified by the (anterior and posterior) insula and its rich projections with the ACC and the orbitofrontal cortex (Fig. 5b) [89, 247–249]. The IFG, embedded within these networks, may encode the certainty of interoceptive inputs and predictions [245]. The structural, functional and effective networks identified in studies of HR and BD cohorts are thus largely those which may support interoception and its visceromotor control through hierarchical (Bayesian) prediction.

Under predictive coding accounts, the psychotic features in SCZ are framed as disturbances to hierarchical inference in exteroception [250]: symptoms as diverse as hallucinosis, abnormal eye movements, sensory attenuation deficits, and delusions are seen as various expressions of the same core pathology, namely, aberrant encoding of the precision of beliefs about the external world [244, 250, 251]. Conversely, mood disorders in general, and BD in particular, may be viewed as a dysfunction in interoceptive inference [252]. Mania reflects a prediction bias towards a rewarding [253], secure, predictable, and “epistemically rich” world [236]. Major depression may result from a ‘locked in’ brain that is relatively insensitive to its (interoceptive) sensory context [239]. Lastly, fatigue is thought to arise from higher-order (“metacognitive”) beliefs regarding the futility of viscero-motor “effort” in response to perceived or real bodily dyshomeostasis [235].

The disturbed subnetworks in BD suggest broad patterns of network dysfunction involving all levels of the interoceptive hierarchy: the symptomatic expression of BD may reflect fronto-limbic dysfunction, that has figured prominently in this review, and which leads to unstable and maladaptive internal representations of the social world. Given the aberrant precision or weighting afforded towards incoming interoceptive signals, features of the external milieu may be consequently perceived in BD patients as increasingly salient, rewarding or threatening [254, 255]. Disturbances in the circuitry of interoceptive centres and regions storing higher-order representations in BD may be sufficient to produce these maladaptive internal models, with the IFG likely prominent in encoding their precision of expected interoceptive sensation. The abnormal activation of the amygdala commonly observed in BD patients may reflect heightened arousal signals due to the resulting dyshomeostasis [239]. Connectivity to cognitive control regions, such as the ACC may embed these predictions in a broader context, such as appraising the self in the current social milieu [171]. Disturbances at these higher hierarchical levels speak to the challenges young HR individuals face regarding affective dysregulation and self-identity. This framework of interoceptive dysregulation can also accommodate the neurovegetative features of BD, particularly the changes in sleep and energy levels. An adaptive failure of this system may also lead to the inability to update these internal models during inter-episode periods, leading to trait cognitive inflexibility [256]. Moreover, fluctuations and compensatory responses on very slow time scales may yield the core “bipolar” hallmark of BD. For example, compensatory responses to the type of connectomic disturbances that figure predominantly in young HR individuals (such as weaker structural and functional connectivity of the AI) may later lead to adaptive (resilience) or mal-adaptive (illness-related) changes in other circuits and other levels of the interoceptive hierarchy.

In closing, we propose BD as a type of “psychosis of interoception”, with unstable neuronal dynamics in corresponding hierarchical systems. The ensuing dysregulation yields the fluctuations in mood at the core of the disorder. The traditional fear circuitry, that include regions such as the amygdala, may not be a primary target of the disorder, but may be maladaptively recruited as a result of misperceptions of threat. This proposed model of abnormal interoceptive inference accommodates the larger-scale patterns of network dysfunction reviewed in this manuscript, and suggests the need for future imaging studies that integrate appropriate emotionally salient tasks with concurrent physiological recordings.
