# Correction: Connectomics of bipolar disorder: a critical review, and evidence for dynamic instabilities within interoceptive networks

**DOI:** 10.1038/s41380-018-0327-7

**Published:** 2019-01-04

**Authors:** Alistair Perry, Gloria Roberts, Philip B. Mitchell, Michael Breakspear

**Affiliations:** 1 QIMR Berghofer Medical Research Institute, Brisbane, QLD Australia; 2 Max Planck UCL Centre for Computational Psychiatry and Ageing Research, Berlin/London, Germany; 3 Center for Lifespan Psychology, Max Planck Institute for Human Development, Lentzeallee 94, 14195 Berlin, Germany; 4 School of Psychiatry, University of New South Wales, Randwick, NSW Australia; 5 Black Dog Institute, Prince of Wales Hospital, Randwick, NSW Australia; 6 Metro North Mental Health Service, Brisbane, QLD Australia

**Keywords:** Neuroscience, Bipolar disorder

**Correction to:** **Molecular  Psychiatry**  (2018).

10.1038/s41380-018-0267-2;published online: 02 October 2018

This Article was originally published under Nature Research's License to Publish, but has now been made available under a [CC BY 4.0] license. The PDF and HTML versions of the Article have been modified accordingly.

